# Case study: effects of low-stress weaning on calf growth performance and carcass characteristics

**DOI:** 10.1093/tas/txad015

**Published:** 2023-02-03

**Authors:** Erin R Gubbels, John R Jaeger, Robin R Salverson, Kristi M Cammack, Judson K Grubbs, Keith R Underwood, Kenneth C Olson, Amanda D Blair

**Affiliations:** Department of Animal Science, South Dakota State University, Brookings, SD 57007; Kansas Agricultural Research Center-Hays, Kansas State University, Hays, KS 67601; Department of Animal Science, South Dakota State University, Brookings, SD 57007; Department of Animal Science, South Dakota State University, Brookings, SD 57007; Department of Animal Science, South Dakota State University, Brookings, SD 57007; Department of Animal Science, South Dakota State University, Brookings, SD 57007; Department of Animal Science, South Dakota State University, Brookings, SD 57007; Department of Animal Science, South Dakota State University, Brookings, SD 57007

**Keywords:** beef, carcass, haptoglobin, low stress, weaning

## Abstract

The objective of this study was to compare the influence of two low-stress weaning methods with conventional weaning on post-weaning performance and carcass characteristics of beef steers. Single-sourced steer calves (*n* = 89) were stratified by body weight (BW) and dam age into three groups in a completely randomized design (*n* = 29 or 30 steers/treatment): ABRUPT (calves isolated from dams on the day of weaning), FENCE (calves separated from dams via a fence for 7 d prior to completely weaning), and NOSE (nose-flap inserted and calves remained with dams for 7 d prior to completely weaning). At day +7 post-weaning, calves were transported to a commercial feedlot where they received standard step-up and finishing rations typical for a Northern Plains feedlot. BWs were recorded in study day −7 (PreTreat), 0 (Weaning), 7 (PostWean), 26 (Receiving), 175 (Ultrasound), and 238 or 268 (Final), and average daily gains (ADG) were calculated for each time period. Blood samples were collected via coccygeal venipuncture at d −7 (PreTreat), 0 (Weaning), and +7 (PostWean) from a subsample of calves (*n* = 10 per treatment) and analyzed for haptoglobin (acute-phase stress protein) concentrations using a bovine haptoglobin ELISA kit. On day 175, ultrasound fat thickness and intramuscular fat were determined and utilized to project marketing dates when steers reached 1.27 cm of backfat (day 238 or 268). Carcass measurements were recorded at the time of harvest. The weaning method interacted (*P* < 0.01) with a time period for ADG and BW. Calf ADG was greater (*P* < 0.01) in the NOSE treatment during PreTreat to Weaning than ABRUPT or FENCE. In the Weaning to PostWean period, the FENCE calves had greater (*P* < 0.01) ADG than ABRUPT and NOSE. During the Postwean to Receiving period ADG was greater (*P* < 0.04) for ABRUPT compared to FENCE and NOSE. Calf ADG was similar (*P* > 0.05) among treatments for the remainder of the feeding period. Calf BW did not differ among treatments (*P* > 0.05) at all times of weighing. Haptoglobin was undetectable in all samples except two samples collected on day −7. The weaning method did not influence (*P* > 0.05) carcass measurements. Collectively these data suggest low-stress weaning methods do not significantly improve post-weaning growth performance or carcass characteristics compared to using conventional methods despite minor, short-term alterations in ADG during the weaning period.

## INTRODUCTION

Weaning is known to be a stressful event for beef cattle. Weaning stress can result in behavioral, hormone, and immune function alterations (Lynch et al., 2012). Acute phase proteins (such as haptoglobin) are stimulated as a defense mechanism in response to trauma, inflammation, or infection (Hughes et al., 2014). Concentrations of acute phase proteins have been shown to be indicators of stress in weaned calves (Arthington et al., 2003). Stress during this time has also been shown to negatively impact calf health and performance (Boland et al., 2008). Therefore, alternative weaning strategies aim to reduce stress at weaning.

Low-stress weaning strategies divide the weaning process into two stages namely 1) physical separation from dams and 2) separation from milk as a nutritional source. It is suggested that two-stage methods decrease the degree of changes in behavior as opposed to simultaneous social and nutritional separation from dams (Haley et al., 2005). Two low-stress strategies that have been utilized in the beef industry include fenceline weaning and the application of anti-suckling devices. Fenceline weaning involves the separation of calves from their dams via a fence such that they reside in adjacent pens or pastures allowing social interaction while preventing suckling (nutritional separation). Anti-suckling devices are inserted into a calf’s nose to prevent suckling but allow contact between the calf and the dam. Research has evaluated the influence of low-stress weaning methods on calf physiology, performance, and health for a short period (days to a few weeks) during and immediately after the weaning process (Haley et al., 2005; Boland et al., 2008; Campistol et al., 2016). Some found short-term stress responses such as reduced gain or increased haptoglobin (stress response), while others did not detect short-term performance or physiological responses to weaning methods (Arthington et al., 2003; Price et al., 2003; Arthington et al., 2005; Haley et al., 2005; Qiu et al., 2007; Arthington et al., 2008; Boland et al., 2008; Campistol et al., 2013; Campistol et al., 2016; Enriquez et al., 2010; Lippolis et al., 2016a). Further, studies investigating the impact of low-stress weaning methods on long-term feedlot performance and carcass characteristics of beef cattle are limited.

At approximately 4– 8 mo of age, new fat cells are forming, and existing cell growth is occurring. This timeframe has been referred to as the marbling “window” by Du et al. (2013). Beef calves are typically weaned during this time period. Stress at this stage could potentially discourage fat cell proliferation and growth and ultimately reduce the amount of intramuscular fat (marbling) present. Reduced marbling scores lead to lower USDA Quality Grades. Therefore, it is plausible that stress incurred during weaning could compromise overall intramuscular fat deposition. We hypothesized that both low-stress weaning methods would improve post-weaning growth performance and carcass characteristics of beef cattle. The objective of this study was to compare the influence of two low-stress weaning methods (fenceline weaning and anti-suckling devices) with conventional abrupt weaning on post-weaning feedlot performance and carcass characteristics of beef steers.

## MATERIALS AND METHODS

### Cattle Management

All animal care and experimental protocols were approved by the South Dakota State University (SDSU) Animal Care and Use Committee (approval number 17-080A). Angus-based steer calves (*n* = 89) from the SDSU Antelope Range and Livestock Research Station near Buffalo, SD were utilized for this study. Angus-based mature cows (4–12 years of age) were mated to two Angus sires during initial artificial insemination. Following artificial insemination, half-sibling Angus bulls were used to complete the breeding season. Steers were stratified by birth date, body weight (BW), and dam age into three groups with one weaning treatment randomly assigned to each group in a completely randomized design (CRD: ABRUPT (calves isolated from dams on the day of weaning, *n* = 29), FENCE (calves separated from dams via a barbed wire fence for 7 d prior to complete separation, *n* = 30), and NOSE (nose-flap inserted and calves remained with dams for 7 d prior to complete separation, *n* = 30). The day of complete separation from dams was the same for all weaning treatments. All cattle resided in one herd from birth until weaning treatments were applied.

At approximately 60 d of age, all steers were vaccinated with a killed vaccine for clostridial diseases (Vision 7 Somnus with SPUR, Merck Animal Health, Madison, NJ). Forty-one days prior to weaning all calves were administered a modified-live vaccine for prevention of bovine rhinotracheitis, bovine viral diarrhea, bovine respiratory syncytial virus Types 1 and 2, and parainfluenza-3 (PI_3_), Haemophilus somnus, and *Mannheimia haemolytica* (Pyramid 5+ Presponse SQ, Boehringer Ingelheim Vetmedica, Inc., St. Joseph, MO). On d −7 (relative to the date of complete separation), steers and dams in FENCE were placed in adjacent pastures separated by a 4-strand barb wire fence. To accomplish this, calves were returned to the pasture that these pairs had been in prior to fenceline separation and dams were placed in the adjacent pasture. Also on d −7, anti-suckling devices (QuietWean, Saskatoon, Saskatchewan, Canada) were inserted in NOSE steers and then steers were allowed to remain with dams until the day of complete separation (day 0). If anti-suckling devices fell out, they were reinserted the same day. During the study, only two devices (93% success rate) fell out and needed to be reinserted (one of the two fell out on the last day). On d 0, anti-suckling devices were removed from NOSE steers and steers from all three treatments were physically separated from their dams. For all treatments, dams were moved to a distant pasture on d 0 to prevent any interaction. Also on d 0, all steers were provided a booster for the clostridial and respiratory disease vaccines and received an anthelmintic (Dectomax Pour-On, Zoetis, Parsippany, NJ). From d 0 to 7, each treatment group of steers was placed in a separate confinement pen and provided ad libitum access to “good” quality grass hay (17.5% CP, 58.3% TDN) in a round-bale feeder and 1.4 kg daily of a commercial weaning supplement (Scranton Equity Exchange, Scranton, ND; 16% CP, 64.9% TDN) in a separate feed bunk. At day 7, all post-weaning calves were transported to a commercial feedlot (Darnall Feedyard, Harrisburg, NE), where all steers were placed in a common pen, received standard step-up and finishing rations (Table 1), and management typical for a Northern Plains feedlot. On day 26 post-weaning all steers were administered a moderate potency initial implant (80 mg trenbolone acetate and 16 mg estradiol; Revalor-IS, Merck Animal Health). On day 175 BW were recorded, steers were administered a high potency finishing implant (200 mg trenbolone acetate and 20 mg estradiol; Revalor-200, Merck Animal Health), and ultrasound fat thickness and intramuscular fat content were measured to predict when steers would reach 1.27 cm fat thickness and utilized to project marketing dates. Cattle were marketed in two groups: the first group (*n* = 42) was marketed at day 238 post-weaning and the second group (*n* = 47) was marketed at day 268 post-weaning. There was similar representation from all treatment groups for both slaughter dates (*n* = 16, 12, and 14 for ABRUPT, FENCE, and NOSE on day 238, respectively; and *n* = 13, 18, and 16 for ABRUPT, FENCE, and NOSE on day 268, respectively). On the day of harvest, steers were transported approximately 166 km to a commercial packing plant.

**Table 1.** Composition of the finishing diet for all steers assigned to different weaning treatments^*a*^

Item	Finishing
Ingredient composition, % of DM	
Dry-rolled corn	58
Sugar beef pulp	20
Dried distiller’s grains with solubles	8
Corn silage	8
Wheat straw	3
Supplement^b^	3
Nutrient composition	
NE_M_, Mcal/kg	2.1
NE_G_, Mcal/kg	1.4
ADF, % of DM	7.4
CP, % of DM	14.2

^*a*^Treatments: ABRUPT: steers abruptly removed from dams in one step (*n* = 29), FENCE: steers separated from dams by a fence for 7 d, then completely separated (*n* = 30), and NOSE: anti-suckling flaps placed in steer noses while with dams for 7 d, then completely separated (*n* = 30)

^*b*^Supplement-contained urea, calcium carbonate, potassium chloride, roughage products, dolomitic limestone, salt, animal fat preserved with ethoxyquin, magnesium oxide, vitamin E supplement, plant protein products, manganese sulfate, zinc sulfate, ferrous sulfate, vitamin A supplement, copper sulfate, calcium iodate, cobalt carbonate, mineral oil, zinc amino acid complex, copper amino acid complex, sodium selenite, and monensin sodium. Monensin included in diet at 30 g/ton.

### Growth Performance and Carcass Characteristics

BW were recorded on study day −7 (PreTreat), 0 (Weaning), 7 (PostWean), 26 (Receiving), 175 (Ultrasound), and 238 or 268 (Final). Measuring BW six times allowed calculation of average daily gain (ADG) for five periods: PreTreat to Weaning, Weaning to PostWean, PostWean to Receiving, Receiving to Ultrasound, and Ultrasound to Final. Carcass measurements were recorded at the time of harvest and included hot carcass weight, ribeye area, 12th rib fat thickness, yield grade, and marbling score.

### Haptoglobin Concentration

While this study focused on post-weaning performance and not weaning period stress, the influence of each weaning method on haptoglobin concentrations during the weaning period was measured as a supplemental analysis to understand if stress may have played a role in post-weaning performance responses. Blood samples were collected via coccygeal venipuncture at day −7 (PreTreat), 0 (Weaning), and +7 (PostWean) from a random subsample of calves (*n* = 10 per treatment). The same subsample of calves was collected at each time point. Blood was allowed to coagulate at room temperature for 1 h and centrifuged at 1,200 × *g* for 30 min at 4 °C. Serum was harvested and stored at −20 °C until analyzed using a bovine haptoglobin enzyme-linked immunosorbent assay (ELISA, Life Diagnostics, INC., West Chester, PA, Catalog Number: Hapt-11) according to manufacturer’s instructions. A plate reader (ELx808; BioTek Instruments, Inc, Winooski, VT) was used to measure optical density at 450 nm. Standard curves for each run of samples were generated using the standards supplied with the ELISA assays. All standard curves had regression coefficients of variation exceeding 0.99. Standard curves were used to back calculate the concentration of haptoglobin in each sample. Haptoglobin was undetectable in all samples except two steers on day −7, rendering further analysis not possible. Haptoglobin concentrations in those two samples were 231.8 and 153.6 µg/mL.

### Statistical Analysis

Because weaning methods were applied to individual calves and the calves resided either with their dams in one pasture-based herd or in a common feedlot pen for most of their lives, calves were considered the experimental unit. Calf ADG and BW were analyzed as a CRD using the Mixed Procedure of SAS 9.4 (SAS Inst., Cary, NC). Weaning treatment, time of measurement, and the treatment by time interaction were considered fixed effects in a factorial treatment structure. Calf ID was considered a random effect. Time was considered a repeated measure and the variance–covariance structure was chosen using Schwarz’s Bayesian Information Criteria goodness of fit statistic. The ante-dependence variance–covariance structure provided the best fit in both ADG and BW analyses. Calf birth BW was included in the model as a covariate for analyses of ADG and BW. Carcass characteristics (hot carcass weight, ribeye area, 12th rib fat thickness, yield grade, and marbling score) were evaluated in a one-way CRD with weaning treatment as a fixed effect and calf ID as a random effect using the MIXED Procedure of SAS. Denominator degrees of freedom were approximated using the Kenward–Roger option in all statistical analyses. When tests for fixed effects were significant at *P* < 0.05, least squares means (lsmeans) and SEM were computed and separated using least significant differences (PDIFF) with a Tukey’s adjustment. For ADG, the *t*-test provided in the SAS output for each lsmean was used to determine if it was different from zero, meaning that the calves neither gained nor lost weight. Responses were considered significant at *P* < 0.05.

## RESULTS AND DISCUSSION

**Figure 1.**
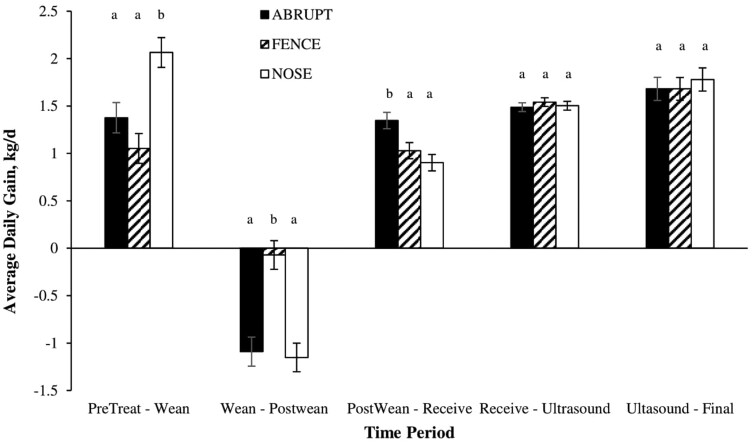
Least squares means for the interaction of weaning treatment^1^ by time period on average daily gain^2^. ^1^ABRUPT (calves isolated from dams on the day of weaning), FENCE (calves separated from dams via a barbed wire fence for 7 d prior to complete weaning), and NOSE (nose-flap inserted and calves remained with dams for 7 d prior to complete weaning). ^2^Average daily gains were calculated for each time period among BWs) recorded on study day −7 (PreTreat), 0 (Weaning), 7 (PostWean), 26 (Receiving), 175 (Ultrasound), and 238 or 268 (Final).^ab^ Bars within each time period lacking a common superscript differ (*P* ≤ 0.05).

**Figure 2.**
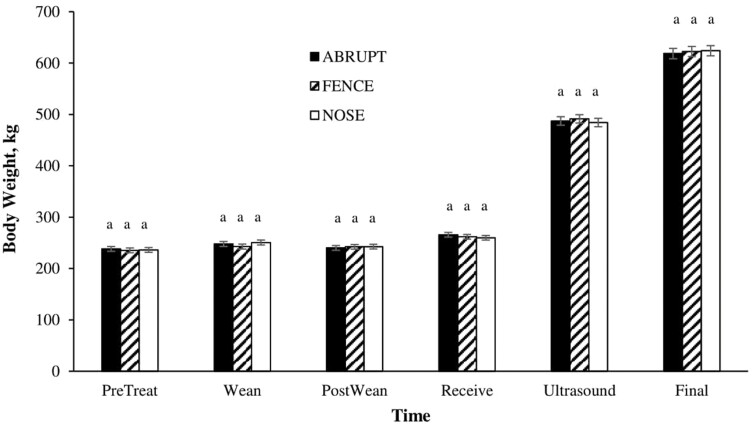
Least squares means for the interaction of weaning treatment^1^ by time period on BW^2^. ^1^ABRUPT (calves isolated from dams on the day of weaning), FENCE (calves separated from dams via a barbed wire fence for 7 d prior to complete weaning), and NOSE (nose-flap inserted and calves remained with dams for 7 d prior to complete weaning). ^2^BWs were recorded on study day −7 (PreTreat), 0 (Weaning), 7 (PostWean), 26 (Receive), 175 (Ultrasound), and 238 or 268 (Final). ^ab^Bars within each time point lacking a common superscript differ (*P* ≤ 0.05).

The weaning method interacted (*P* < 0.01) with the time period for ADG and BW (Figures 1 and 2, respectively). The average daily gain was greater (*P* < 0.01) for calves in the NOSE treatment during the PreTreat to Wean period than for calves in ABRUPT and FENCE, while ABRUPT and FENCE calves had similar (*P* > 0.05) ADG (Figure 1). This suggested that physical separation by a fence negatively affected ADG compared to NOSE calves which were prevented from suckling but had a close association with dams. During the Wean to PostWean period, the FENCE calves had ADG that was not different (*P* > 0.05) than zero (maintained BW) but were greater (*P* < 0.01) than the similar but negative ADG of ABRUPT and NOSE calves (−1.09, −0.07, and −1.15 ± 0.154 kg, for ABRUPT, FENCE, and NOSE, respectively). This indicated the FENCE calves had begun to adapt to separation from their dams, allowing them to maintain weight, while the ABRUPT and NOSE calves experienced weight loss during this period. These findings were similar to results by Boland et al. (2008) where calves subjected to fenceline weaning gained more BW 7 d post-weaning, while nose-flap calves lost weight. Boland et al. (2008) also observed the fenceline and abrupt groups had increased BW gains the week prior to weaning compared to the nose-flap group. However, Haley et al. (2005) reported greater ADGs one-week post-weaning in calves weaned using nose-flaps. During the PostWean to Receive period in the current study, ADG was greater (*P* < 0.04) for the ABRUPT treatment compared to the FENCE and NOSE treatments, with FENCE and NOSE being similar (*P* > 0.05). Campistol et al. (2013) and Enriquez et al. (2010) also reported that weight gains during this time period were greater in calves that were abruptly weaned compared to calves that were fenceline weaned. In contrast, Price et al. (2003) reported that calves weaned using the fenceline weaning method gained more weight up to 10 wk post-weaning than calves weaned abruptly. Further, Enriquez et al. (2010) found calves weaned using the fenceline method had greater BW gains compared to the noseflap method, which contrasts with the findings in the current study that ADG was similar during this period among the two low-stress weaning methods.

It has been reported that decreased ADG and BW could be explained by increased time walking, standing, and vocalizing instead of more time eating, laying down, and ruminating (Haley et al., 2005). Although behavior was not analyzed in the present study, it could provide an explanation for BW and average daily gain responses that were observed. In the present study, treatment did not influence (*P* > 0.05) ADG during the Receive to Ultrasound or Ultrasound to Final time periods, indicating that the weaning method did not influence performance beyond the receiving period. Other studies (Freeman et al., 2021; Lippolis et al., 2016a) that followed calves through finishing reported similar feedlot performance results to the current study, although Bailey et al. (2016) found greater ADG in fenceline vs. abrupt weaned calves because fenceline calves were compensating for reduced ADG during the receiving and growing phases.

There was a treatment-by-time interaction (*P* < 0.01) for BW. Mean separation indicated BW was similar across treatments at each weigh time (Figure 2), despite differences in ADG during the pre-and post-weaning periods. In contrast, Campistol et al. (2013) found calves weaned using fenceline weaning had increased BW one-week post-weaning when compared to calves abruptly weaned, while Lippolis et al., (2016a) reported that at up to 21 d post-weaning, abruptly weaned calves tended to weigh more than calves weaned using noseflaps.

The weaning method did not influence (*P* > 0.05) hot carcass weight, ribeye area, 12th rib fat thickness, yield grade, or marbling score (Table 2). This lack of influence on carcass traits suggested that differences in stress experienced around the weaning event were not significant enough to cause long-term changes in carcass composition. Particularly, because marbling scores were similar between treatments, potential stress experienced by calves during the weaning event was not adequate to cause alterations in intramuscular fat deposition. Other studies (Bailey et al., 2016; Lippolis et al., 2016b; Freeman et al., 2021) that followed calves through finishing also reported no effect of weaning methods on carcass responses.

**Table 2.** Least squares means for the fixed effect of weaning treatment on carcass characteristics

Variable	ABRUPT^*a*^	FENCE^*a*^	NOSE^*a*^	SEM^*b*^	*P* value^*c*^
Hot carcass weight, kg	387	390	389	6.5	0.94
Ribeye area, cm^2^	87.42	87.61	89.42	1.88	0.70
Twelvth rib fat thickness, cm	1.37	1.40	1.55	0.071	0.12
Yield grade	3.13	3.20	3.27	0.114	0.71
Marbling score^*d*^	504	541	512	18.5	0.33

^*a*^Treatments: ABRUPT: steers abruptly removed from dams in one step (*n* = 29), FENCE: steers separated from dams by a fence for 7 d, then completely separated (*n* = 30), and NOSE: anti-suckling flaps placed in steer noses while with dams for 7 d, then completely separated (*n* = 30).

^*b*^Standard error of the mean.

^*c*^Probability of a greater *F* for test of weaning treatment fixed effect.

^*d*^Marbling score: 400 = Small^0^, 500 = Modest^0^, 600 = Moderate^0^.

Haptoglobin was undetected in all but two samples collected on day −7. While haptoglobin concentration has been reported to increase as a result of trauma or stress (Hughes et al., 2014), it has been suggested that haptoglobin is not easily detected at basal levels and is almost undetectable in cattle that are not experiencing stress (Arthington et al., 2003). These results suggest that the calves in this study may not have experienced detectable stress during weaning regardless of treatment. However, the differences in ADG among treatments during various portions of the weaning process suggest otherwise.

## CONCLUSION

This case study is limited in that there was only one group replicate per treatment, with individuals within treatment groups evaluated as experimental units. This limitation influences the rigor of conclusions that may be drawn. Despite this potential limitation, the authors suggest low-stress weaning methods reduce performance during the weaning stress period, but they do not significantly influence overall post-weaning growth performance or carcass characteristics compared to calves weaned using conventional methods. It may be efficacious for producers to take into consideration and implement low-stress weaning methods for improved performance at weaning or during early backgrounding phases if calves are to be marketed during those time periods. Alternatively, if calves will not be marketed during or immediately after weaning, these data suggest producers can expect overall performance and economic returns to be unaffected by weaning method. More research is warranted with additional replicates to confirm these findings.
